# Consequences of omitting additional treatment after local excision of high-risk early rectal cancer: a national cohort

**DOI:** 10.1007/s00384-025-05052-z

**Published:** 2026-01-03

**Authors:** William Lossius, Tore Stornes, Tor Åge Myklebust, Arne Wibe

**Affiliations:** 1 Department of Surgery, St. Olav’s Hospital, Trondheim University Hospital, Trondheim, Norway; 2 Norwegian Research Center for Minimally Invasive and Image-Guided Diagnostics and Therapy, St. Olav’s Hospital, Trondheim University Hospital, Trondheim, Norway; 3 Institute of Clinical and Molecular Medicine, Norwegian University of Science and Technology, Trondheim, Norway; 4 Department of Registration, Cancer Registry of Norway, Norwegian Institute of Public Health, Oslo, Norway; 5 Department of Research and Innovation, Møre and Romsdal Hospital Trust, Ålesund, Norway

**Keywords:** Local excision, TEM, TAMIS, Early rectal cancer

## Abstract

**Purpose:**

This study aimed to evaluate oncological outcomes in patients with high-risk early rectal cancer undergoing local excision, comparing those who received guideline-recommended additional treatment to those who did not, either due to comorbidities or personal preference.

**Method:**

National data on patients treated by transanal endoscopic microsurgery (TEM) or transanal minimally invasive surgery (TAMIS) for early rectal cancer without prior chemoradiotherapy between 2010 and 2020 were analyzed retrospectively. Patients were classified into low-risk (pT1 without risk factors for lymph node involvement) and high-risk (pT1 with risk factors and all pT2). High-risk patients receiving additional treatment (mainly completion TME, or less frequently adjuvant chemoradiotherapy for high-risk pT1) were compared to those without further treatment. Endpoints were 5-year relative survival, disease-free survival, overall survival, local recurrence, and distant recurrence.

**Results:**

Among 298 patients, 70 (23.5%) were low-risk pT1, 153 (51.3%) were high-risk pT1, and 75 (25.2%) were pT2. Additional treatment was omitted in 93 (60.8%) of high-risk pT1 and 39 (52.2%) of pT2 cases. Compared to patients following guidelines, those not receiving additional treatment had lower 5-year disease-free survival of 53.3% vs. 80.9% (*p* = 0.008) and higher 5-year local recurrence rates of 22.0% vs. 7.3% (*p* = 0.008). Five-year overall survival was 63.9% vs. 90.6% (*p* = 0.013), and relative survival 81.9% vs. 97.7% (*p* = 0.157).

**Conclusion:**

Omitting indicated additional treatment following TEM or TAMIS for high-risk early rectal cancer is associated with a substantially higher local recurrence rate and loss of long-term disease-free survival and overall survival.

## Introduction

### Background/rationale

Local excision (LE) by transanal endoscopic microsurgery (TEM) or transanal minimally invasive surgery (TAMIS) is an alternative to total mesorectal excision for the treatment of early rectal cancer. In selected patients, it may reduce complications and improve functional outcomes, without compromising survival [1–3]. The introduction of colorectal screening programs in many countries, Norway included, is expected to increase the rate of early-stage rectal cancer by about one-third, highlighting the need for refined treatment strategies [4, 5]. Early rectal cancer (ERC) can be defined as a tumor confined to the submucosa or muscularis propria of the rectal wall (T1-2N0M0), and subdivided based on the risk of lymphovascular spread into low-risk pT1, high-risk pT1, and pT2 cancer.

According to Norwegian guidelines, LE by TEM or TAMIS can be offered to pT1 tumors < 3 cm without high-risk features such as deep submucosal infiltration (sm3 or > 1000 µm, or Haggitt > 3), low/poor differentiation, involved margins (< 1 mm), lymphovascular invasion, or moderate to high-grade tumor budding [6]. Completion surgery with total mesorectal excision (cTME) is advised to patients where the histopathological report reveals such characteristics, and recently, the Norwegian guidelines have approved the use of adjuvant chemoradiotherapy (aCRT) as an alternative to cTME in the case of high-risk T1 tumors.

Moreover, for the elderly and frail, with increased risk of mortality and morbidity from formal resection, LE with or without aCRT may be a compromise strategy in high-risk early rectal cancer. Furthermore, patients fit for major surgery may defer recommended completion surgery, accepting increased risk of local recurrence and possibly reduced survival to avoid major surgery, especially in the case of stoma formation. Data are scarce regarding the need for additional treatment following local excision of ERC, and the consequences of omitting such treatment.

The challenges in preoperative diagnostics and selection of patients for LE, as well as the potentially increased risk of permanent stoma formation in cTME vs. direct mesorectal excision, have been addressed in a previous paper [7].

A meta-analysis from 2020 estimated the rates of local and distant recurrence for low-risk pT1 to be about 7% and 3%, respectively. Significantly higher local recurrence rates were found for both high-risk pT1 and pT2 tumors without additional treatment. Both aCRT and cTME seemed to give acceptable results for high-risk pT1 tumors, while cTME seemed necessary to achieve comparable results for pT2 tumors. Distant recurrence rates for high-risk pT1 and pT2s were 4–7% without significant difference between the strategies [8]. A systematic review from 2018 suggested new recurrence rates after salvage surgery (i.e., surgery performed for local recurrence after LE) to be 3% for local recurrence, but 13% for crude overall disease recurrence and estimated a 5-year disease-free survival of about 50%, and that poor prognosis due to distant metastasis seems to be a challenge despite achieving local control with salvage surgery [9]. Both studies concluded that there is a need for more data on the outcome of local excision with or without additional treatment [8, 9].

### Objectives

The aim of this study was to evaluate the oncological outcomes of patients undergoing local excision for high-risk early rectal cancer with additional treatment according to guidelines, compared to patients who do not undergo indicated additional treatment, due to either compromise for comorbidity or personal preference. The secondary aims of this study were to estimate the probability of additional treatment after local excision for early rectal cancer, as well as the oncological outcome of recurrence following local excision.

## Methods

### Study design

This was a retrospective analysis of national registry data.

### Setting

The study was conducted at St. Olav’s University Hospital in Trondheim, Norway, and the Cancer Registry of Norway.

### Participants

All patients treated for early rectal cancer, defined as pathological T1-2N0M0 (stage I), treated by local excision by transanal excision, TEM or TAMIS (Nordic Medico-Statistical Committee Classification of Surgical Procedures (NSCP) codes JGA73 Transanal excision of lesion of rectum, JGA75 Endoscopic microsurgical excision of lesion of rectum, JGA82 Transanal endoscopic resection of submucosa, JGA85 Transanal endoscopic full-thickness excision), in Norway during the time period of 1 January 2010 to 31 December 2020 were included. Histological report of both LE and following formal rectal resections were revised manually by WL and TS, for selection of T1 into low-risk and high-risk and for identification of the following exclusion criteria:

- histological types other than adenocarcinoma (e.g., neuroendocrine tumors), or pathologic review revealing T3-stage
- conventional/open transanal excision
- endoscopic snare resections as well as piecemeal endoscopic mucosal resections (EMR), unless followed up by local excision by TEM or TAMIS
- concurrent colon cancer
- neoadjuvant therapy prior to LE

### Variables

The following variables were collected:

- Baseline characteristics: date of diagnosis and of surgery, age, sex, type of surgery, and distance of tumor from anal verge, TNM stage, submucosal infiltration, differentiation, lymphovascular infiltration, tumor budding, and resectional margins.
- Data on further treatment: time to radiotherapy, time to and histology report of cTME or salvage surgery (TNM stage of recurrence, resectional margins, multiviscerality of resection).
- Data on outcomes: date to local recurrence, distant recurrence, and death.

### Data sources/measurement

The Norwegian Colorectal Cancer Registry (NCCR) is part of the Cancer Registry of Norway. The NCCR prospectively collects data on all colorectal cancers in Norway and has a high level of completeness [10]. Clinicians involved in the treatment of colorectal cancers are committed to reporting data on work-up, treatment, and follow-up.

### Follow-up

According to national guidelines, follow-up entailed serum carcinoembryonic antigen and rigid proctoscopy every 6 months, and either abdominal ultrasound or CT at 6 and 18 months, and a thoracoabdominopelvic CT at 12, 24, 36, 48, and 60 months. Patients > 75 years at diagnosis, or patients at high risk of recurrence, may have had individualized adaptations to this program.

### Definitions

Local recurrence: tumor recurrence in the rectal wall or pelvic node after four months following LE. If discovered within 4 months, it was defined as remaining/incompletely removed tumor.

Distant metastasis: metachronous metastasis occurring after 4 months following LE (if within 4 months, it was considered synchronous).

cTME: completion TME surgery performed within four months of LE, based on unfavorable histopathologic report of LE.

Salvage surgery: TME surgery performed after four months following LE, for local recurrent disease.

aCRT, adjuvant chemoradiotherapy: chemoradiotherapy started within four months of LE.

Neoadjuvant chemoradiotherapy: for patients included in this study, this refers to chemoradiotherapy started after four months following LE and prior to salvage surgery.

### Statistical methods

Categorical variables were described as frequencies and percentages, and continuous variables were summarized as means. For survival analyses, patients were sorted into the following three groups: A, low-risk pT1 treated by local excision alone; B, high-risk pT1 followed up with either cTME or aCRT, or pT2 with cTME (according to national guidelines); C, high-risk pT1 or pT2 without additional recommended treatment (i.e., not treated according to guidelines). Patients were followed from the date of LE until the date of the event of interest (death from all causes, local recurrence, or distant recurrence) or the date of administrative censoring (31 December 2022), whichever came first. Disease-free survival (DFS) was estimated by the Kaplan-Meier analysis, defining local recurrence, distant metastasis, or death by any cause as events. Relative survival was estimated using the Pohar-Perme estimator [11]. Cumulative incidence of local recurrence and distant metastases was estimated using the Aalen-Johansen estimator, treating death from any cause as a competing risk. Cox proportional hazards regressions were used to estimate unadjusted hazard ratios (HR), as well as HRs adjusted for age and sex for all outcomes. For relative survival, we estimated unadjusted and adjusted excess hazard ratios (EHR) using flexible parametric excess hazard models using stpm3 [12]. Stata/SE 18.0 for Windows (StataCorp LLC, College Station, TX, USA) was used for statistical analysis.

## Results

We identified 407 patients from the NCCR database, and after manually reviewing the histological reports for exclusion criteria, 298 remained for analysis. Median follow-up was 4.8 years (IQR 2.3–7.2) overall, and 6.4 years (IQR 4.4–8.9) for patients without events (death or any recurrence) (see Tables 1 and 2 for baseline characteristics and treatment based on risk groups).

**Table 1:** Baseline characteristics

	Low-risk pT1	High-risk pT1	pT2
TEM/TAMIS (*n* = 298)	70 (23.5%)	153 (51.3%)	75 (25.2%)
Age	67.5 (SD 12.0)	70.8 (SD 11.8)	74.0 (SD 11.1)
Sex, male	58.6%	55.6%	56.0%
Risk factor^a^			
Deep submucosal infiltration		134 (87.6%)	
Lymphovascular infiltration		30 (19.6%)	
Tumor budding		4 (2.6%)	
Poor differentiation		8 (5.2%)	
Involved or uncertain margin		34 (22.2%)	
Deep submucosal infiltration as sole risk factor		91 (59.5%)	

^a^A high-risk pT1 case may have more than one risk factor

**Table 2:** Patients by risk group and additional therapy

Treatment	Risk group			Total
	pT1 low-risk	pT1 high-risk	pT2	
No additional treatment	69 (98.6%)	93 (60.8%)	39 (52.2%)	201 (67.5%)
cTME	0	57 (37.3%)	29 (38.7%)	86 (28.9%)
aCRT	1 (1.4%)	3 (2.0%)	7 (9.3%)	11 (3.7%)
Total	70 (100%)	153 (100%)	75 (100%)	298 (100%)

Among the 86 cTME procedures, residual disease was found in the bowel wall only in 11 patients (12.8%), in mesorectal lymph nodes only in 10 patients (11.6%), and in both sites in 3 patients (3.5%), totaling 24 patients (27.9%).

**Fig. 1:**
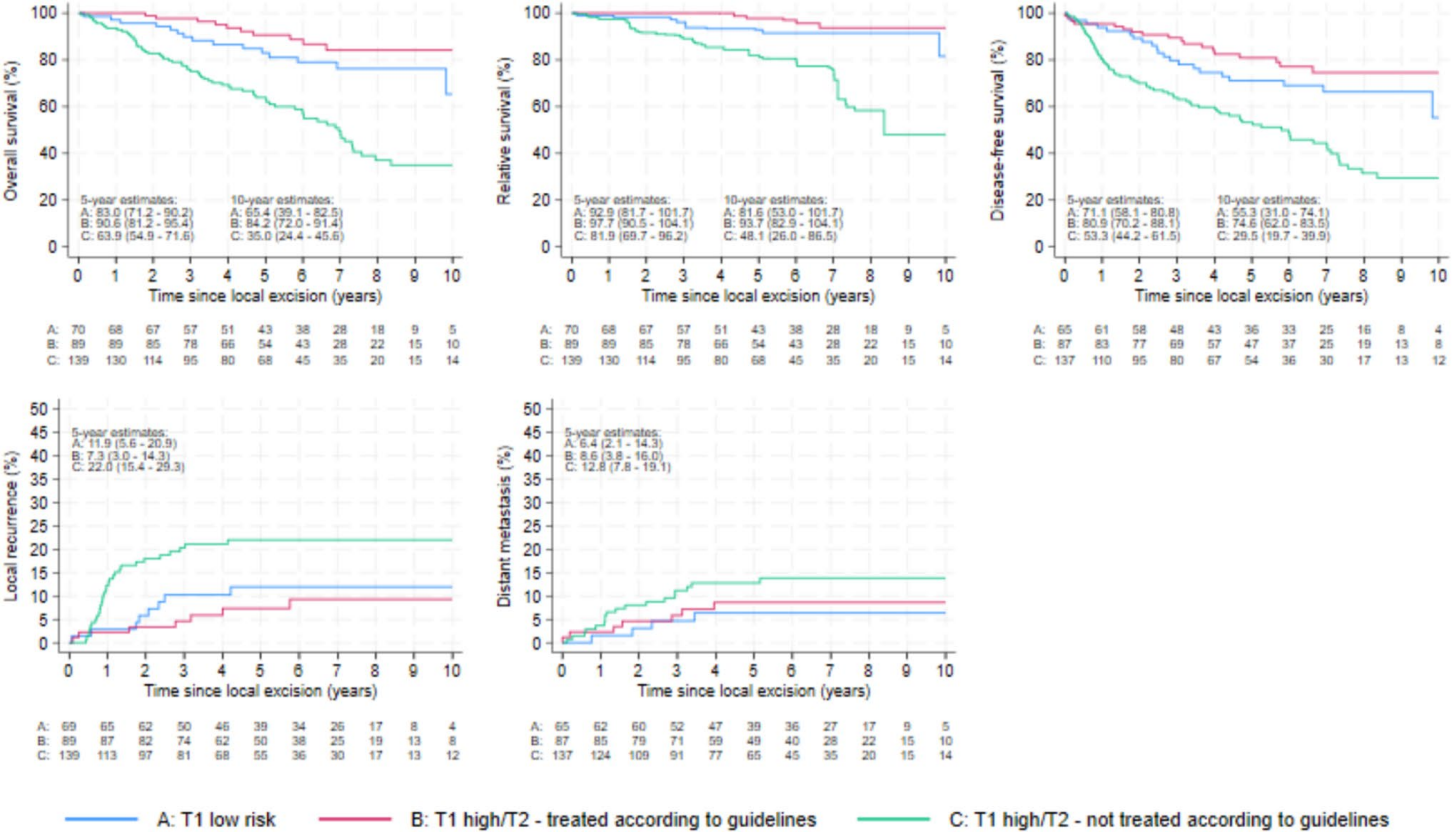
Oncological results by risk group and treatment. Blue line (A): low-risk pT1 treated by local excision alone; red line (B): high-risk pT1 followed up with either cTME or aCRT, or pT2 with cTME (according to national guidelines); green line (C): high-risk pT1 or pT2 without additional recommended (i.e., not treated according to guidelines)

Overall survival for group B (high-risk pT1 followed with either cTME or aCRT, or T2 with cTME) was 90.6% (95% CI 81.2–95.4) vs. 63.9% (95% CI 54.9–71.6) for group C (high-risk pT1 or pT2 without additional recommended treatment) at 5 years, and 84.2% (95% CI 72.0–91.4) vs. 35.0% (95% CI 24.4–45.6) at 10 years (Fig. 1). Relative survival for group B was 97.7% (95% CI 90.5–104.1) vs. 81.9% (95% CI 69.7–96.2) for group C at 5 years, and 93.7% (95% CI 82.9–104.1) vs. 48.1% (95% CI 26.0–86.5) at 10 years. Disease-free 5-year survival for group B was 80.9% (95% CI 70.2–88.1) vs. 53.3% (95% CI 44.2–61.5) for group C, and 74.6% (95% CI 62.0–83.5) vs. 29.5% (95% CI 19.7–39.9) at 10 years. At 5 years, local recurrence was 7.3% (95% CI 3.0–14.3) for group B vs. 22.0% (95% CI 15.4–29.3) for group C, and distant recurrence was 8.6% (95% CI 3.8–16.0) vs. 12.8% (7.8–19.1), respectively.

Among the 91 patients with deep submucosal infiltration as the sole risk factor, 56 received no additional treatment, and the 5-year estimated local recurrence for this group was 10.8% (95% CI 4.4–20.5), and the 5-year estimated disease-free survival was 67.7% (95% CI 53.1–78.7%).

Multivariable analysis showed significant differences for group C compared to group B (adjusted for age and sex) for overall survival (HR 2.47 [95% CI 1.12–5.02], *p* = 0.013), disease-free survival (HR 2.11 [95% CI 1.22–3.65], *p* = 0.008), and local recurrence (HR 3.23 [95% CI 1.37–7.64], *p* = 0.008). Results of all uni- and multivariable analyses are summarized in Table 3.

**Table 3:** Univariable and multivariable analyses of oncological outcomes

	Overall survival (HR [95% CI], *p*)	Relative survival (HR [95% CI], *p*)	Disease-free survival (HR [95% CI], *p*)	Local recurrence (HR [95% CI], *p*)	Distant metastasis (HR [95% CI], *p*)
Group A (unadjusted)	1.93 (0.87–4.30), 0.107	1.99 (0.00–13051.59), 0.878	1.52 (0.81–2.85), 0.195	1.53 (0.56–4.22), 0.410	0.77 (0.23–2.63), 0.676
Group A (adjusted)^a^	1.14 (0.63–3.20), 0.39	0.00, 0.998	1.28 (0.68–2.41), 0.453	1.51 (0.55–4.19), 0.442	0.77 (0.22–2.62), 0.676
Group B	1	1	1	1	1
Group C (unadjusted)	5.48 (2.82–10.66), **0.000**	32.77 (0.04–29505.12), 0.315	3.44 (2.06–5.75), **0.000**	3.36 (1.47–7.65), **0.004**	1.85 (0.77–4.43), 0.167
Group C (adjusted)^a^	2.47 (1.21–5.02), **0.013**	15.46 (0.35–687.09), 0.157	2.11 (1.22–3.65), **0.008**	3.23 (1.37–7.64), **0.008**	1.87 (0.74–4.72), 0.187

^a^Adjusted for age and sex; A, low-risk pT1 treated by local excision alone; B, high-risk pT1 followed up with either cTME or aCRT, or pT2 with cTME (according to national guidelines); C, high-risk pT1 or pT2 without additional recommended (i.e., not treated according to guidelines). Bold values indicate statistical significance (p < 0.05)

### Outcomes following local recurrence

**Fig. 2:**
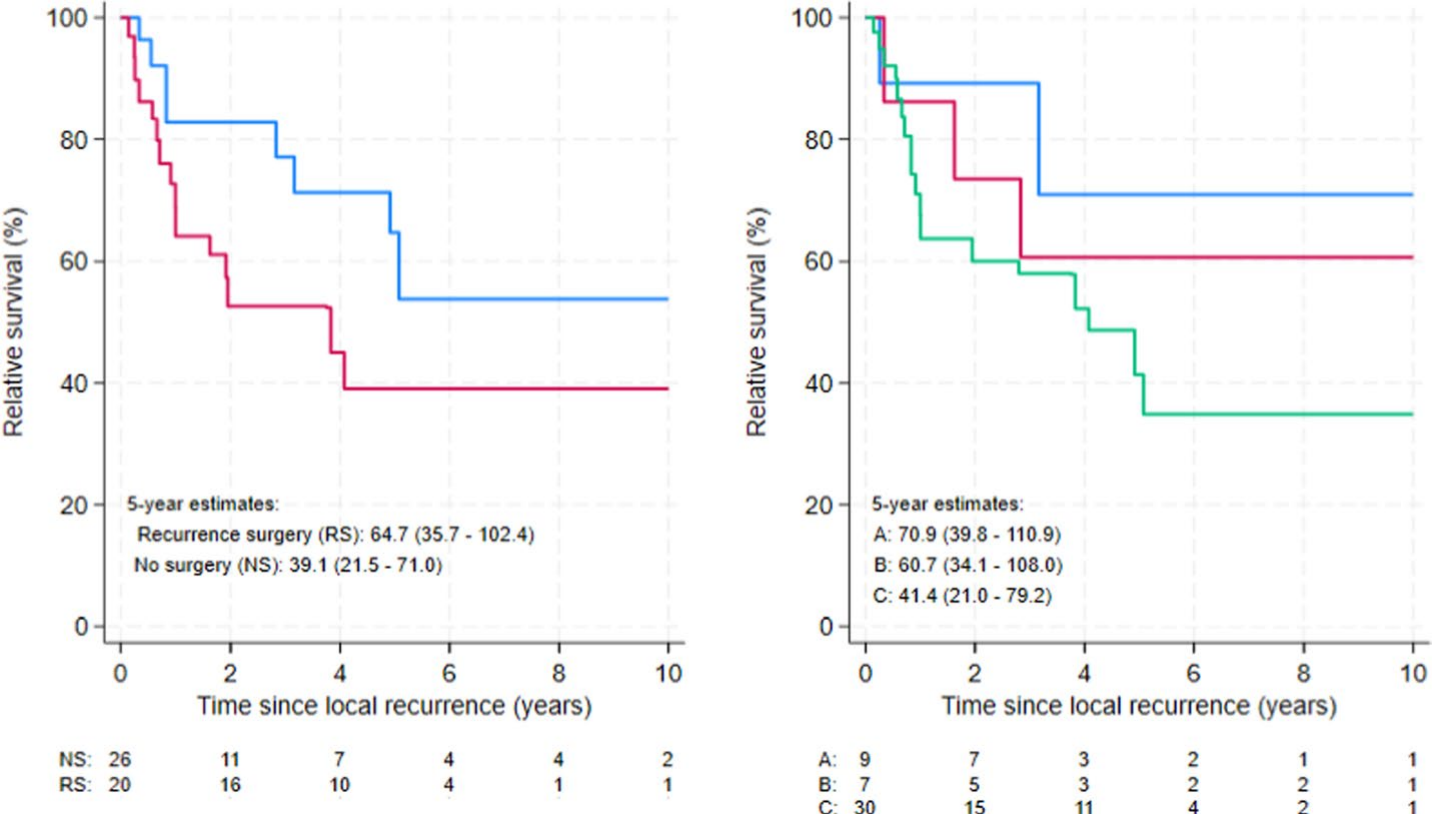
Relative survival following recurrence after local excision. Blue line: recurrence surgery (RS). Red line: no recurrence surgery (NS). Blue line (A): pT1 low risk. Red line (B): pT1 high risk/pT2 treated according to guidelines. Green line (C): pT1 high risk/pT2 without additional guideline indicated treatment

In the 298 patients, there were 46 cases of recurrence (15.4%), and 20 of these (43.5%) underwent salvage TME surgery for recurrence. Among the 46 patients with recurrence, ten were diagnosed with synchronous distant metastasis, and nine developed metachronous metastasis within the follow-up. Two of the salvage procedures (10.0%) involved multivisceral surgery and 15 (75.0%) achieved an R0 resection. Among the 20 salvage TME patients, one had synchronous metastasis at the time of recurrence, another was diagnosed with metastasis within 4 months, and five more developed metastases at a later point. Five-year distant metastasis for patients undergoing salvage TME was 30.5% (95% CI 10.9–52.9). Figure 2 shows relative survival, stratified by salvage surgery status and by initial risk groups A, B, and C, compared with the age- and sex-matched general population.

## Discussion

The main finding of this study was that the group with ERC not treated according to guidelines suffered a loss of both disease-free and overall 5-year survival of about 28% and 27%, respectively. This compromise strategy was chosen either due to patient preference or frailty, but the cost is substantial and needs to be taken into consideration when advising patients eligible for cTME or adjuvant chemoradiotherapy.

Comparable to a meta-analysis from 2020 [8], the present compromise group experienced three-fold more local recurrences, 22% vs. 7.3%, than those receiving additional treatment, as well as some more distant metastases, 12.8% vs. 8.6%, both being responsible for reduced disease-free survival.

The 5-year relative survival of groups A and B (93% and 98%, respectively) was comparable to the 99% 5-year relative survival of all patients undergoing resectional surgery for stage I rectal cancer in Norway in the same period [10]. Overall, disease-free and relative survival continued to decrease towards 10 years for group C, while local recurrence and distant metastasis remained unchanged at ten years compared to at 5 years, suggesting that loss of survival after 5 years was mainly due to direct or indirect causes related to local recurrences and distant metastases developed within the first 5 years.

Only about a quarter (23.49%) of the patients undergoing local excision for early rectal cancer were classified as low-risk pT1 cancers where no additional treatment was indicated. Based on histopathological criteria and the guidelines that were in use during the study period, three-quarters of the patients should have been recommended completion TME. However, in spite of national guideline recommendations, about 60% of high-risk pT1 tumors and more than half (52.2%) of the pT2 tumors did not receive additional treatment. This suggests either widespread use of local excision as a compromise in patients unfit for major surgery, or a high degree of patient preference to abstain from cTME.

The low proportion of radiotherapy for high-risk patients in this cohort reflects that aCRT was not part of the national treatment program during the study period. As the new national guidelines endorse treating high-risk pT1 tumors with either aCRT or cTME, about three in four patients treated with local excision for early rectal cancer today can be expected to be treated with an organ-sparing approach, provided that aCRT is used in preference to cTME for high-risk pT1 tumors. This might negate the possible concerns of increased risk of a permanent stoma due to the need for additional treatment following transanal procedures for early rectal cancer compared to primary TME.

About 60% of the high-risk pT1 tumors had deep submucosal infiltration (DSI) as the sole risk factor. A recent meta-analysis on risk factors of lymph node metastasis in pT1 colorectal cancer did not find DSI alone as a significant independent predictor, and the absolute risk was found to be 2.6%. It concluded that DSI alone should be reconsidered as an indication for further oncologic therapy [13]. In the current study, patients in this subgroup who did not receive additional treatment seemed to have comparable local recurrence and disease-free survival to the low-risk group. While this is in contrast to the conclusions of other studies [14, 15], further evidence concluding on this matter might theoretically reclassify 60% of the high-risk pT1 tumors into low-risk, potentially reducing the need for additional treatment.

Five-year relative survival for local recurrence after local excision was 64.7% for those offered salvage surgery compared to 39.1% for those who did not undergo such surgery. The risk group stratification after local excision seemed to be associated with differences in relative survival after diagnosis of recurrence as well, with 5-year estimates at 60.7% for group B compared to 41.1% for group C. This might reflect the compromise group where extensive additional treatment was not feasible, but with the limited number of cases with recurrence, detailed oncological outcome analysis, comparing risk groups and effect of salvage surgery, was not deemed feasible. However, the poor relative survival for all groups does seem to support previous evidence of waiting for local recurrence before performing TME surgery in high-risk locally excised ERC to be a poor strategy [9].

### Strenghts and weaknesses

This study is retrospective and does not allow for accurate differentiation between compromise patients unfit for major surgery and patient preference for organ preservation. The differences in oncological results between groups B and C need to be interpreted with some degree of caution, as our dataset does not allow for adjustment for comorbidities in analyzing outcomes. On the other hand, the study presents real-world data at a national level of the use of and results of local excision for early rectal cancer, quality assured by manual revision of all pathological reports. Regarding results of salvage TME, re-recurrence locally is not reported as reliably as distant metastasis, and thus we were not able to estimate re-recurrence or disease-free survival after salvage TME.

## Conclusion

Omitting indicated additional treatment following TEM or TAMIS for high-risk early rectal cancer is associated with a substantially higher local recurrence rate and loss of long-term disease-free survival and overall survival. Based on histopathological criteria and current guidelines, about three out of four patients undergoing local excision for early rectal cancer might be treated with an organ-sparing approach by local excision alone or in conjunction with aCRT.

## Data Availability

The data that support the findings of this study are not openly available due to reasons of sensitivity and are available from the corresponding author upon reasonable request. Data are located in controlled access data storage at the Cancer Registry of Norway, Norwegian Institute of Public Health, Oslo, Norway.

## References

[1] Clermonts S, van Loon YT, Wasowicz DK, Langenhoff BS, Zimmerman DDE (2018) Comparative quality of life in patients following transanal minimally invasive surgery and healthy control subjects. J Gastrointest Surg 22(6):1089–1097 29508218 10.1007/s11605-018-3718-9

[2] Restivo A, Zorcolo L, D’Alia G, Cocco F, Cossu A, Scintu F et al (2016) Risk of complications and long-term functional alterations after local excision of rectal tumors with transanal endoscopic microsurgery (TEM). Int J Colorectal Dis 31(2):257–266 26298182 10.1007/s00384-015-2371-y

[3] Hompes R, Ashraf SQ, Gosselink MP, van Dongen KW, Mortensen NJ, Lindsey I et al (2015) Evaluation of quality of life and function at 1 year after transanal endoscopic microsurgery. Colorectal Dis 17(2):O54-61 25476189 10.1111/codi.12858

[4] Logan RF, Patnick J, Nickerson C, Coleman L, Rutter MD, von Wagner C (2012) Outcomes of the Bowel Cancer Screening Programme (BCSP) in England after the first 1 million tests. Gut 61(10):1439–1446 22156981 10.1136/gutjnl-2011-300843 PMC3437782

[5] Holme Ø, Løberg M, Kalager M, Bretthauer M, Hernán MA, Aas E et al (2018) Long-term effectiveness of sigmoidoscopy screening on colorectal cancer incidence and mortality in women and men: a randomized trial. Ann Intern Med 168(11):775–782 29710125 10.7326/M17-1441 PMC6853067

[6] Helsedirektoratet (2022) Nasjonalt handlingsprogram med retningslinjer for diagnostikk behandling og oppfølging av kreft i tykktarm og endetarm. 9th ed. Available from: https://www.helsedirektoratet.no/retningslinjer/kreft-i-tykktarm-og-endetarm-handlingsprogram?tidligere-versjoner#48656784. Accessed 9 Dec 2025

[7] Lossius WJ, Stornes T, Myklebust TA, Endreseth BH, Wibe A (2022) Completion surgery vs. primary TME for early rectal cancer: a national study. Int J Colorectal Dis 37(2):429–435 34914000 10.1007/s00384-021-04083-6 PMC8803686

[8] van Oostendorp SE, Smits LJH, Vroom Y, Detering R, Heymans MW, Moons LMG et al (2020) Local recurrence after local excision of early rectal cancer: a meta-analysis of completion TME, adjuvant (chemo)radiation, or no additional treatment. Br J Surg 107(13):1719–1730 32936943 10.1002/bjs.12040 PMC7692925

[9] Jones HJS, Cunningham C, Nicholson GA, Hompes R (2018) Outcomes following completion and salvage surgery for early rectal cancer: a systematic review. Eur J Surg Oncol 44(1):15–23 29174708 10.1016/j.ejso.2017.10.212

[10] Kreftregisteret (2024) Årsrapport 2023 Resultater og forbedringstiltak fra Nasjonalt kvalitetsregister for tykk- og endetarmskreft. Available from: https://www.kreftregisteret.no/Registrene/Kvalitetsregistrene/Tykk-ogendetarmskreftregisteret. Accessed 9 Dec 2025

[11] Perme MP, Stare J, Estève J (2012) On estimation in relative survival. Biometrics 68(1):113–120 21689081 10.1111/j.1541-0420.2011.01640.x

[12] Lambert P (2023) STPM3: stata module to fit flexible parametric survival models. Statistical software components S459207, Boston College Department of Economics, revised 95 Dec 2024. Available from: https://ideas.repec.org/c/boc/bocode/s459207.html. Accessed 9 Dec 2025

[13] Zwager LW, Bastiaansen BAJ, Montazeri NSM, Hompes R, Barresi V, Ichimasa K et al (2022) Deep submucosal invasion is not an independent risk factor for lymph node metastasis in T1 colorectal cancer: a meta-analysis. Gastroenterology 163(1):174–189 35436498 10.1053/j.gastro.2022.04.010

[14] Bosch SL, Teerenstra S, de Wilt JH, Cunningham C, Nagtegaal ID (2013) Predicting lymph node metastasis in pT1 colorectal cancer: a systematic review of risk factors providing rationale for therapy decisions. Endoscopy 45(10):827–834 23884793 10.1055/s-0033-1344238

[15] Ebbehøj AL, Jørgensen LN, Krarup PM, Smith HG (2021) Histopathological risk factors for lymph node metastases in T1 colorectal cancer: meta-analysis. Br J Surg 108(7):769–776 34244752 10.1093/bjs/znab168

